# Characterization of acetate transport in colorectal cancer cells and potential therapeutic implications

**DOI:** 10.18632/oncotarget.12156

**Published:** 2016-09-21

**Authors:** Suellen Ferro, João Azevedo-Silva, Margarida Casal, Manuela Côrte-Real, Fatima Baltazar, Ana Preto

**Affiliations:** ^1^ CBMA- Centre of Molecular and Environmental Biology, Department of Biology, University of Minho, Campus de Gualtar, Braga, Portugal; ^2^ ICBAS - Institute of Biomedical Sciences Abel Salazar, University of Porto, Porto, Portugal; ^3^ Life and Health Sciences Research Institute (ICVS), School of Health Sciences, University of Minho, Braga, Portugal; ^4^ ICVS/3B's - PT Government Associate Laboratory, Braga/Guimarães, Portugal

**Keywords:** colorectal cancer, monocarboxylate transporters, short-chain fatty acids, acetate, 3-bromopyruvate

## Abstract

Acetate, together with other short chain fatty acids has been implicated in colorectal cancer (CRC) prevention/therapy. Acetate was shown to induce apoptosis in CRC cells. The precise mechanism underlying acetate transport across CRC cells membrane, that may be implicated in its selectivity towards CRC cells, is not fully understood and was addressed here. We also assessed the effect of acetate in CRC glycolytic metabolism and explored its use in combination with the glycolytic inhibitor 3-bromopyruvate (3BP). We provide evidence that acetate enters CRC cells by the secondary active transporters MCT1 and/or MCT2 and SMCT1 as well as by facilitated diffusion *via* aquaporins. CRC cell exposure to acetate upregulates the expression of MCT1, MCT4 and CD147, while promoting MCT1 plasma membrane localization. We also observed that acetate increases CRC cell glycolytic phenotype and that acetate-induced apoptosis and anti-proliferative effect was potentiated by 3BP. Our data suggest that acetate selectivity towards CRC cells might be explained by the fact that aquaporins and MCTs are found overexpressed in CRC clinical cases. Our work highlights the importance that acetate transport regulation has in the use of drugs such as 3BP as a new therapeutic strategy for CRC.

## INTRODUCTION

Colorectal cancer (CRC) is one of the most common cancers and cause of cancer death in developed countries, highlighting the need of novel strategies for prevention/therapy of CRCs [1].

Short chain fatty acids (SCFA), namely acetate, propionate and butyrate are produced by bacterial fermentation of dietary fiber that escape absorption in the small intestine. These compounds are produced in a millimolar ratio of approximately 60:20:20, respectively [2], being a major source of energy for colonocytes. It was shown that *in vivo* administration of *Propionibacterium freudenreichii* significantly increased apoptosis in colon cells damaged with a carcinogenic agent (1,2-dimethylhydrazine) without affecting the survival of healthy normal colonocytes [3, 4]. We and others, previously established that acetate affects CRC cells survival *in vitro* [5–9]. We showed that acetate inhibits CRC cell proliferation, induces apoptosis, promotes lysosomal membrane permeabilization with release of cathepsin D, which is associated with an autophagy-independent degradation of damaged mitochondria [5, 9]. The reason for acetate selectivity towards transformed colon cells without affecting normal colon cells is still elusive.

To exert their cellular effect, SCFA must be transported across the plasma membrane [10]. SCFA (including acetate) can either enter normal colon cells through passive diffusion or by membrane transporters mainly monocarboxylate transporter-1 (MCT1) and sodium-coupled monocarboxylate transporter SMCT1 [1, 11]. In CRC cells, the majority of the reports studied butyrate transport and showed that MCT1 is the main implicated transporter [1, 12, 13]. However, the precise mechanism of acetate transport in CRC cells has not been characterized and might contribute to its selectivity to CRC cells.

MCT overexpression has been described in several cancer types, including CRC, being involved in the maintenance of glycolytic metabolism by mediating lactate export [14, 15]. MCTs have been explored as therapeutic targets [16] and as mediators of the entry of drugs such as the anticancer compound 3-bromopyruvate (3BP) [14, 17].

Since acetate is the most relevant SCFA produced in the colon, although less studied, we aimed herein to characterize the mechanism of acetate transport across the plasma membrane of CRC cells. We also intended to evaluate the effect of acetate on glycolytic metabolism, as well as to explore the use of acetate in combination with 3BP as a novel therapeutic strategy in CRC.

## RESULTS

### Kinetics and energetics of acetate transport by colorectal cancer cells

**Figure 1:**
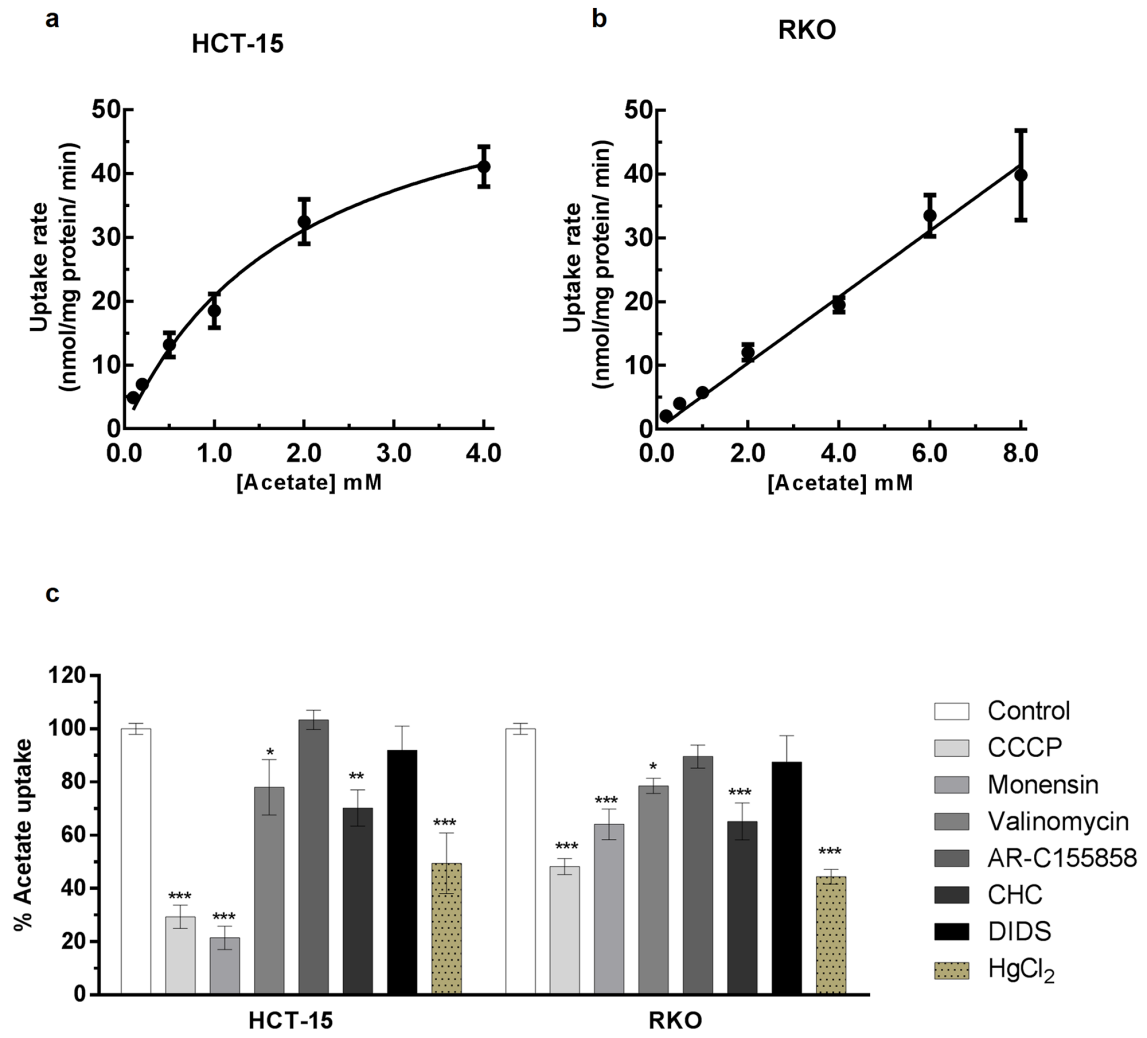
Characterization of acetate uptake in CRC cells **a, b.** Plots of the initial uptake rates of labeled acetate, as a function of the acid concentration at pH 6.0 in HCT-15 (a) and RKO (b). Kinetic parameters, as affinity constant (K_m_) and transport capacity (V_max_) or Diffusion constant (K_d_) for the uptake of acetate were based on the non-linear regression for the Michaelis-Menten equation f [V]=(Vmax x [Acetate])/(Km + [Acetate]) and Passive diffusion equation f[V] = Kd.[Acetate]. In HCT-15 the transporter system shows a K_m_ of 1.97 ± 0.57 mM and a V_max_ of 62 ± 9 nmol/ mg of protein/ min. In RKO acetate enters cells by passive diffusion with a K_d_ of 5.19 ± 0.16. **(c)** Effect of CCCP (100 μM), Monensin (100 μM), Valinomycin (100 μM), AR-C155858 (1 μM), CHC (10 mM), DIDS (1 mM) and HgCl_2_ (100 μM) in the uptake of 1 mM of acetate. Statistical analysis was performed by two-way ANOVA: ***, ** and * indicate significant differences with a respective P-values of <0.001, <0.01 and <0.05 (n=3).

The initial uptake rates of [^14^C] acetate were evaluated in HCT-15 and RKO cell lines at pH 6.0 (Figure 1a and 1b). The analysis of non-linear regression showed that in HCT-15 cells, acetate transport follows a second order kinetics with an affinity constant (*K_m_*) of 1.97 ± 0.57 mM and a transport capacity (*V_max_*) of 62 ± 9 nmol/mg of protein/min. In RKO cells, acetate transport follows a first order kinetics with a diffusion constant of 5.19 ± 0.16 μL/mg of protein/min.

To evaluate the energetics of [^14^C] acetate transport we tested the influence of agents known to disrupt different ion membrane electrochemical potentials such as CCCP (a protonophore which disrupts both the proton and the electrical gradient), monensin (an ionophore that specifically disrupts sodium gradient) and valinomycin (an ionophore which preferentially affects potassium and sodium gradient across biological membranes). We also tested the inhibition of acetate transport using inhibitors of monocarboxylate transporters such as CHC (inhibitor of MCT1 and SMCT1) [18, 19], DIDS (inhibitor of MCTs and anion exchangers) [20] and AR-C155858 (inhibitor of MCT1 and MCT2) [21] (Figure 1c).

Our results showed that CCCP inhibited significantly acetate transport in both HCT-15 (71%) and RKO (52%) cells; monensin also inhibited acetate uptake in HCT-15 (78%) and RKO (36%), and valinomycin presented only a small inhibitory effect in both cell lines (22% and 21% in in HCT-15 and RKO cells, respectively). The results obtained with CCCP inhibitory effects led us to conclude that acetate transport in CRC cells is sensitive to the plasma membrane potential. Moreover, monensin inhibition indicates that sodium gradient is also important in this process. Concerning valinomycin inhibition, the transport of acetate is less affected suggesting that potassium gradient might not be involved but rather its effect might be due to the disruption of the sodium gradient, taking into account the results with monensin.

Regarding general MCTs inhibitors: CHC inhibited both HCT-15 (30%) and RKO cells (35%), while DIDS and AR-C155858 had no inhibitory effect suggesting a possible contribution of at least SMCT1 in acetate uptake in CRC cells.

**Figure 2:**
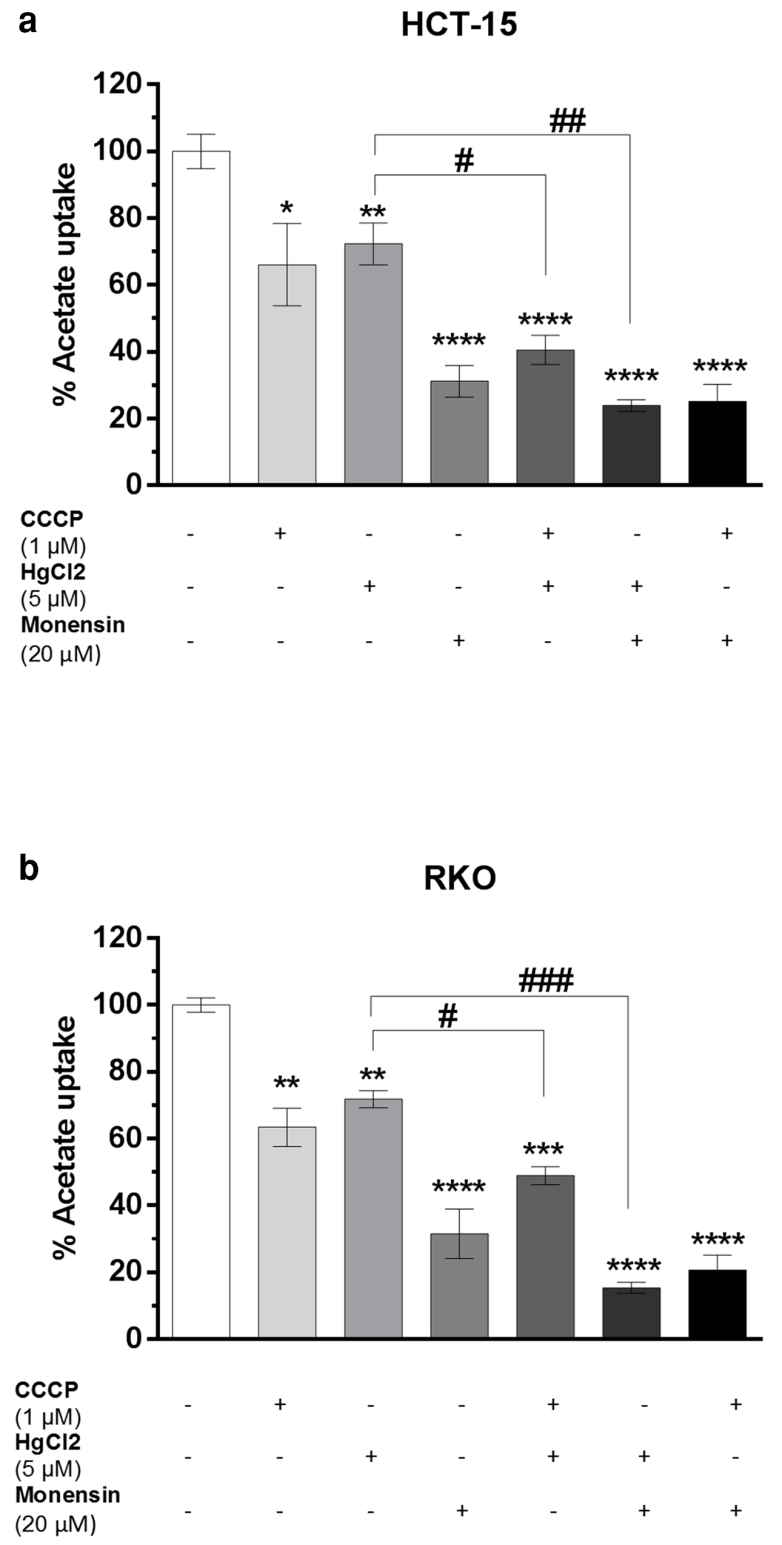
Inhibition of 1 mM of acetate uptake by CCCP (1 μM), HgCl_2_ (5 μM) and Monensin (20 μM) alone or in combination in HCT-15 a and RKO b. cells. Statistical analysis was performed by two-way ANOVA (n=3): **** ***, ** and * indicate significant differences with a respective P-values of <0.0001 <0.001, <0.01 and <0.05 compared to negative control and ###, ##, # indicates P-values of <0.001, <0.01 and <0.05 compared to HgCl_2_ inhibition alone with combination (HgCl_2_ with CCCP and HgCl_2_ with Monesin).

**Figure 3:**
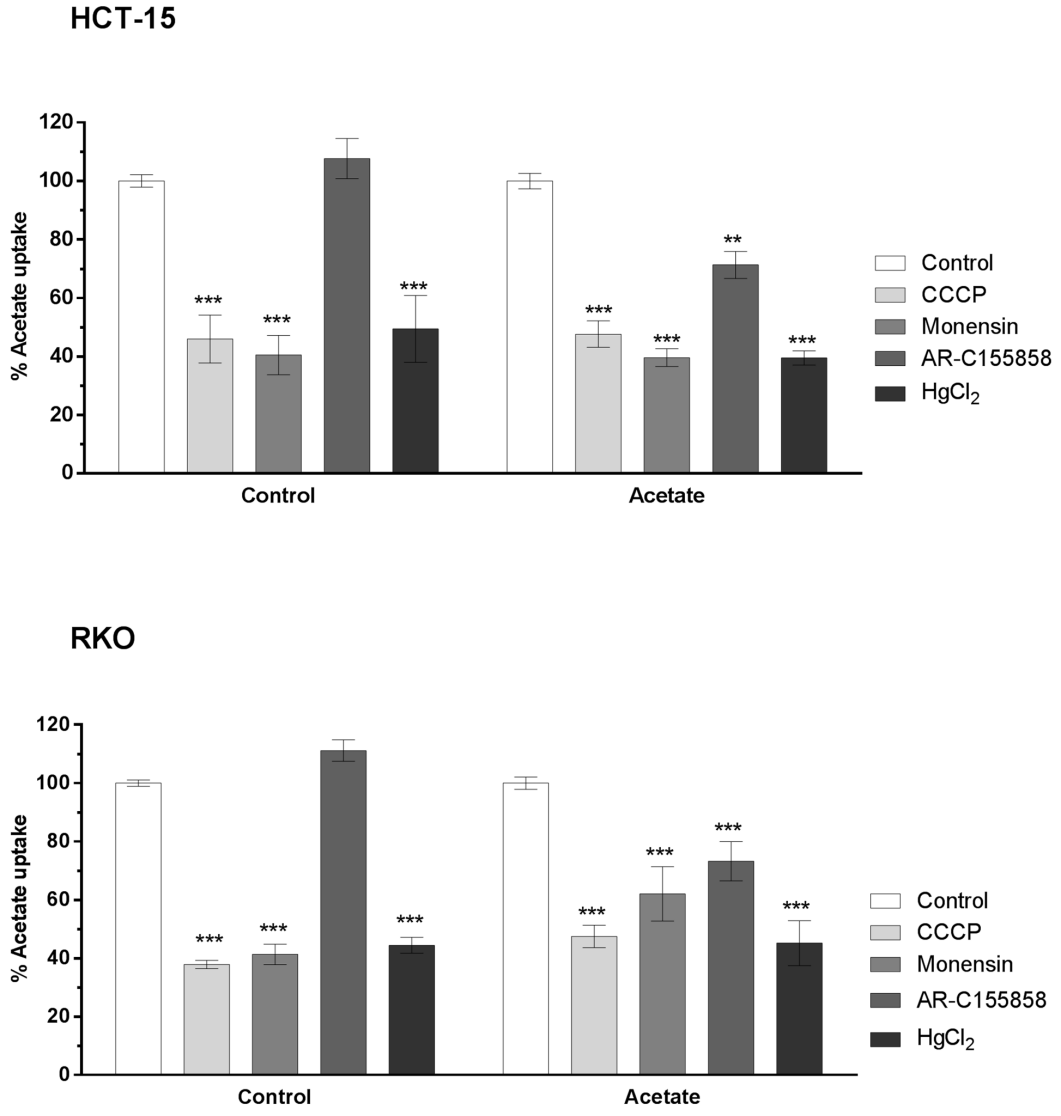
Inhibition of acetate (1 mM) uptake in HCT-15 and RKO cell lines treated with IC_30_ of acetate for 48h with CCCP (100 μM), Monensin (100 μM), AR-C155858 (1 μM) and HgCl_2_ (100 μM) Statistical analysis was performed by two-way ANOVA: ***, ** and * indicate significant differences with a respective P -values of <0.001, <0.01 and <0.05 (n=3).

The transport of acetate in normal colon cells has also been shown to occur through passive transport [11]. This transport component may be relevant in RKO cells, since acetate transport followed a first order kinetics and was less affected by CCCP and monensin than HCT-15 cells. A possible contribution of aquaporins to acetate uptake could also explain the acetate kinetics in RKO cells. Aquaporins are small transmembrane channel proteins that allow the passage of water and other small solutes, such as glycerol and some ions, through cell membranes in response to osmotic gradient [22, 23]. Furthermore, aquaporin 1 and aquaporin 3 are upregulated in CRC cells and their expression was correlated with tumor growth, invasiveness and metastasis [22, 24]. Consequently, we used HgCl_2_, which is described as an inhibitor of aquaporin activity (especially of certain classes such as aquaporin 1 and aquaporin 3) [23, 25] to verify if acetate transport into CRC cells could also be mediated through passive transport by aquaporins. Interestingly, HgCl_2_ inhibited acetate uptake in HCT-15 (51%) and RKO (56%) cells, suggesting the contribution of aquaporins to acetate transport in both CRC cell lines (Figure 1c). We further investigated if inhibition of acetate transport by HgCl_2_ in HCT-15 and RKO cells was independent of the inhibition detected for CCCP or monensin. To this end we assess the effect of the combined used of the inhibitors. However, in order to avoid saturation and enable the evaluation of the combined effect of the different drugs, the concentrations of the inhibitors used in these combination experiments were lower than the concentrations used in the experiments were they were tested alone (Figures 1 and 3). Our results showed that while the use of CCCP (1 μM), HgCl_2_ (5 μM) and monensin (20 μM) (Figure 2a) caused inhibition of acetate transport in HCT-15 cells of 34%, 37%, and 69%, respectively, the dual combination of CCCP/HgCl_2_, HgCl_2_/monensin, and CCCP/monensin resulted in an inhibition of 56%, 76% and 75%, respectively.

In RKO cells (Figure 2b) acetate uptake inhibition by CCCP, HgCl_2_ and monensin was 37%, 28% and 68%, respectively, but when CCCP/HgCl_2,_ or monensin/ HgCl_2_ were combined acetate transport inhibition was 46% and 81%, respectively.

Altogether these results point to the involvement of a secondary active transport dependent on the electrochemical Na^+^ gradient (probably involving SMCT1) and a passive transport by a facilitated diffusion component mediated through aquaporins in CRC cells.

### Acetate upregulates the expression of MCT1, MCT4 and CD147 in colorectal cancer cells

We further assessed the kinetics and energetics of acetate uptake in cell cultures exposed with levels of acetate mimicking its intestinal microenvironment concentration. To test this hypothesis, HCT-15 and RKO cells were exposed for 48 hours to IC_30_ and IC_50_ doses of acetate previously determined by us [5].

Upon incubation with IC_30_ during 48 hours we could observe that acetate transport, in contrast to cells non-exposed to acetate, was significantly inhibited by AR-C155858 in HCT-15 and RKO cells (29% and 23%, respectively). This specific inhibitory effect suggests the involvement of MCT1 and/or MCT2 in acetate uptake under these conditions. In addition, the inhibitory pattern for CCCP, monensin and HgCl_2_ was not altered (Figure 3). The relative values of acetate transport were expressed as percentage in relation to the control (without inhibitor) in each condition. Moreover, no significant alteration in acetate uptake capacity was observed between cells treated and untreated with acetate (data not shown).

**Figure 4:**
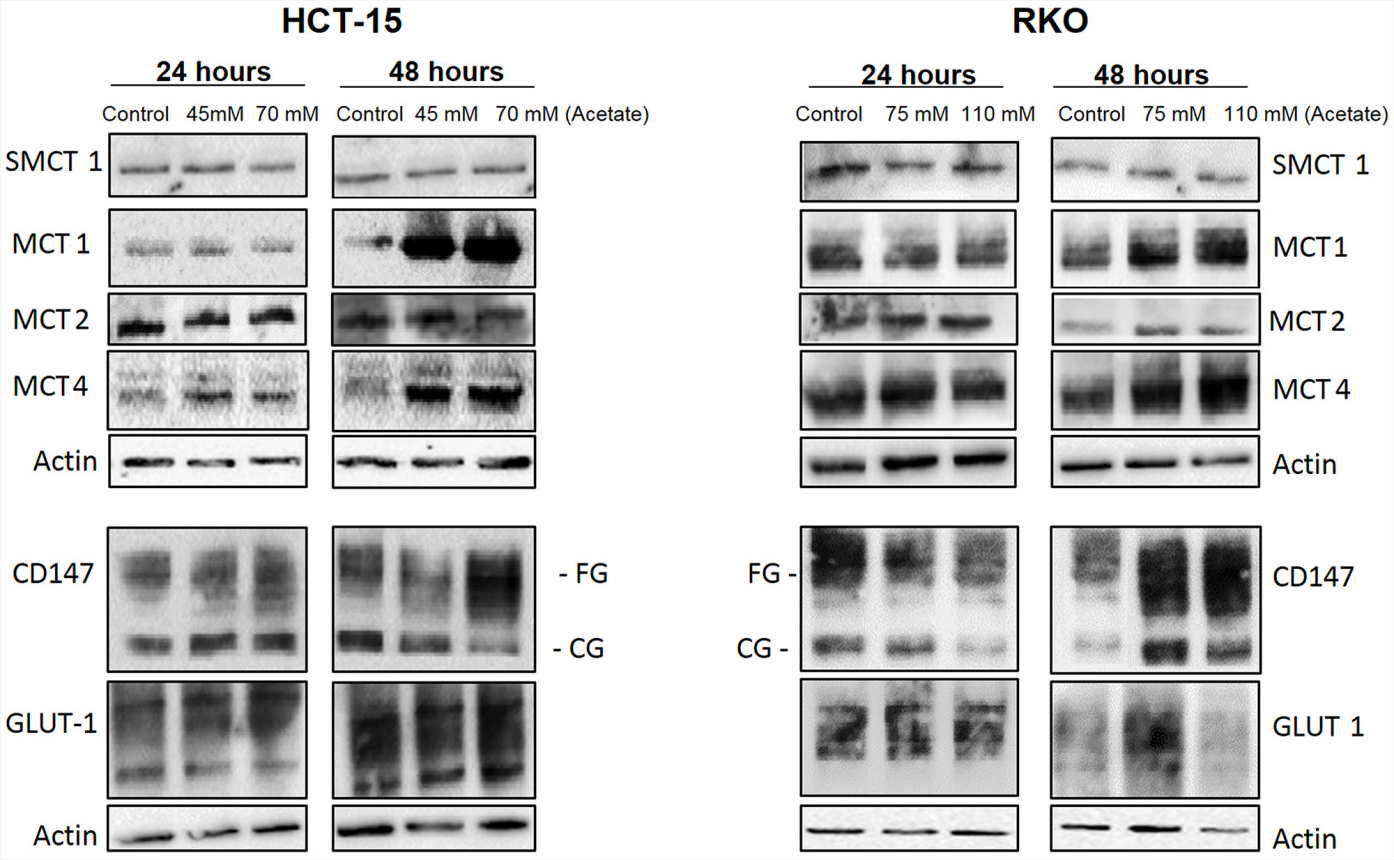
Characterization of MCTs expression and other glycolytic markers in CRC cells after acetate treatment HCT-15 and RKO cells were incubated with acetate (45 mM and 70 mM for HCT-15 cells; 75 mM and 110 mM for RKO cells, respective doses for the IC_30_ and IC_50_) or with fresh medium (as negative control) for 24 and 48 hours. Western blotting images of the SMCT1, MCT1, MCT4, MCT2, CD147 and GLUT-1 expression. β-actin was used as loading control. A representative experiment of at least three independent experiments is shown.

We further assessed the expression profile of SMCT1, MCT1, MCT4, MCT2 and of the chaperone cluster of differentiation CD147 (an important protein for MCT activity, including MCT1 and MCT4) in response to acetate (Figure 4). Extracts of CRC cells exposed to IC_30_ and IC_50_ acetate doses for 24 and 48 hours, were analyzed by Western blot. We found that the expression of SMCT1 and MCT2 was not affected by exposure to acetate. In contrast the expression of MCT1 and MCT4 was enhanced only after 48 hours, in both cell lines compared to the negative control. Furthermore, the chaperone CD147, was present in both fully-glycosylated (FG) and core-glycosylated (CG) forms in both CRC cell lines after 48 hours of acetate treatment. We have also observed that in both cells the expression of GLUT-1 transporter, responsible for basal glucose transport [26], was not affected. Overall our results suggest that in CRC cells acetate upregulates the expression of MCT1, MCT4 and CD147.

### Acetate induces MCT1 plasma membrane localization in colorectal cancer cells

**Figure 5:**
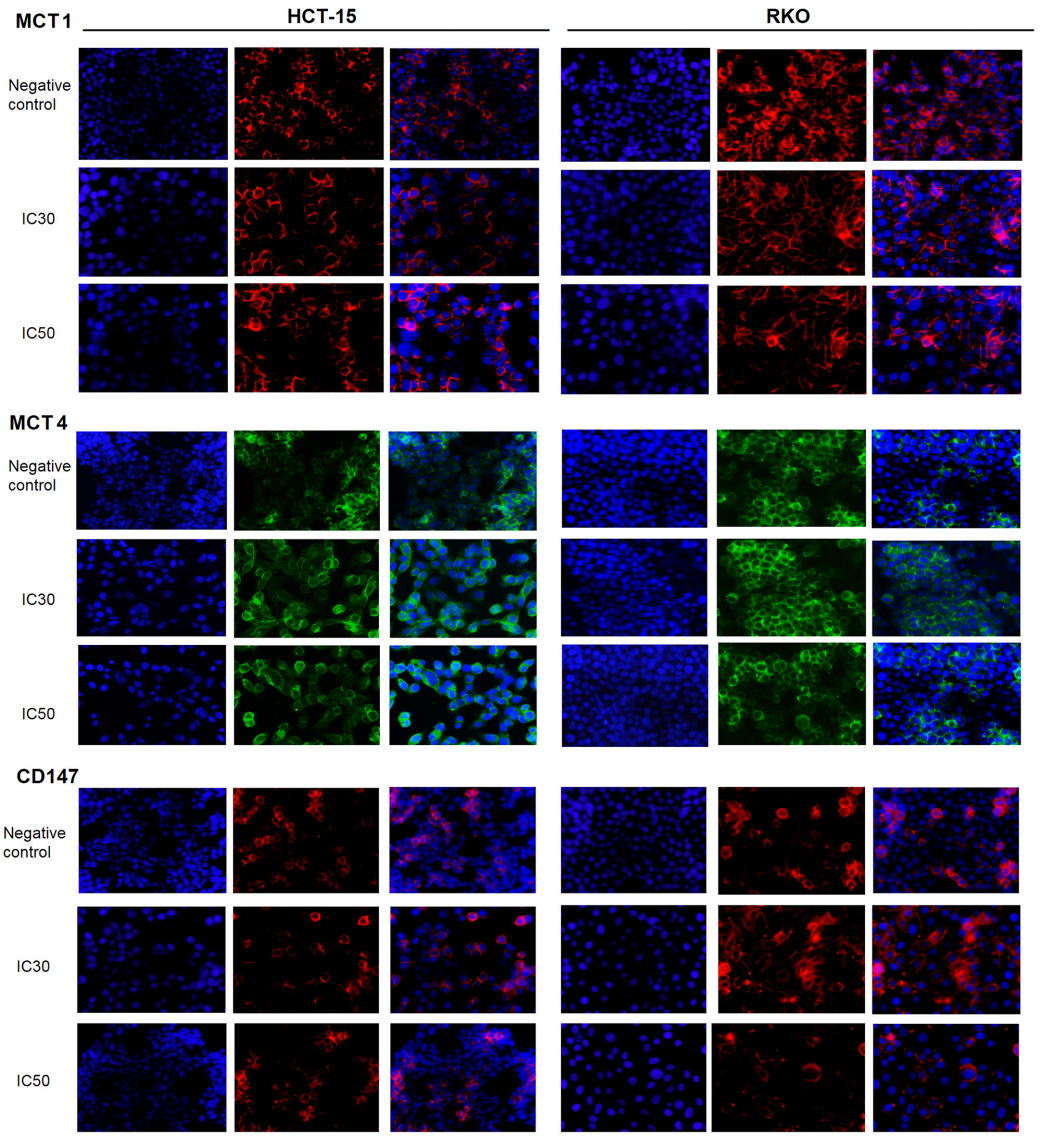
Localization of MCT1, MCT4 and CD147 in CRC cells after acetate treatment HCT-15 and RKO cells were incubated with acetate (45 mM and 70 mM for HCT-15 cells; 75 mM and 110 mM for RKO cells, respective doses for the IC_30_ and IC_50_) or with fresh medium (as negative control) for 48 hours. Representative images of immunofluorescence are shown (400× magnification).

We further analyzed by immunofluorescence the changes induced by acetate on the cellular localization of MCT1, MCT4 and CD147. We could observe that acetate leads to an elevated expression of these transporters and an increase in MCT1 localization at the plasma membrane in comparison to the cytoplasm in both cell lines (Figure 5). Moreover, we found no differences between MCT4 and CD147 co-localization after acetate treatment. Our results suggest that in the presence of acetate, at concentrations similar to those observed in the colon, there is an increase in MCT1 localization at the plasma membrane, which might enhance the membrane transport of acetate by MCT1 in CRC cells.

### Acetate treatment perturbs the glycolytic metabolism and 3-bromopyruvate potentiates acetate-induced apoptosis in colorectal cancer cells

**Figure 6:**
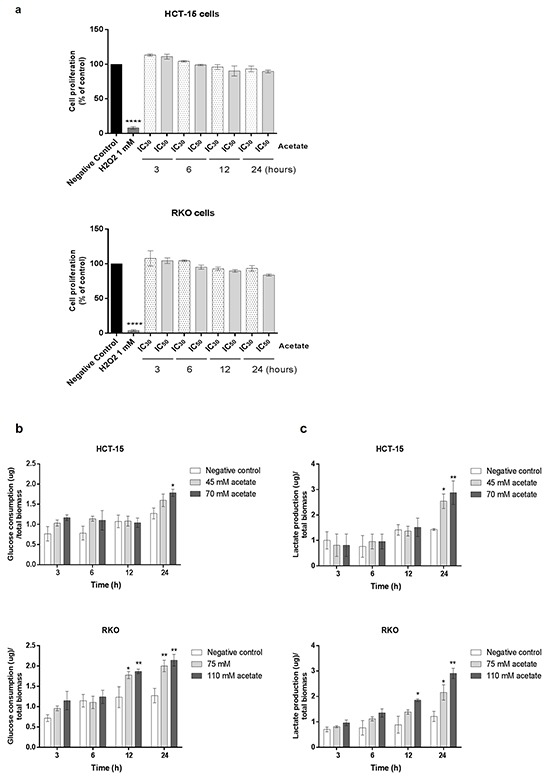
Acetate induces metabolic changes in CRC cells HCT-15 and RKO cells were incubated with acetate (45 mM and 70 mM for HCT-15 cells; 75 mM and 110 mM for RKO cells, respective doses for the IC_30_ and IC_50_) or with fresh medium (as negative control). **a.** Effect of acetate on cell proliferation, determined by SRB assay. HCT-15 and RKO cells were seeded at a density of 7x10^4^ and 5x10^4^ cells/well respectively and incubated with sodium acetate (3, 6, 12 and 24 hours). As positive control was used 500 uL and 1 mM H_2_O_2_, respectively for HCT-15 and RKO cells. As negative control cells were incubated with fresh complete medium. For each bar, the mean for at least three independent experiments is represented (Bonferroni's test; *** p ≤ 0.001 compared to control cells). **b.** Extracellular amounts of glucose consumption and **c.** lactate production overtimes 3, 6, 12 and 24 hours are shown. Values are expressed as mean ± SD of at least three independent experiments. *P ≤ 0.05 and **P ≤ 0.01 compared with control cells.

We therefore wondered if acetate might induce metabolic changes. HCT-15 and RKO cells were exposed to IC_30_ and IC_50_ doses of acetate and the relative rate of glucose consumption and extracellular lactate production were measured at 3, 6, 12 and 24 hours in triplicate and normalized to cell biomass determined by SRB at T0 (Figure 6a). None of these conditions showed alteration in cell proliferation. We found that CRC cells in response to acetate exhibited a higher consumption of glucose (p > 0.05) and lactate production rates (p > 0.05; p > 0.01) up to 24 hours in a dose-dependent manner (Figure 6b, 6c).

**Figure 7:**
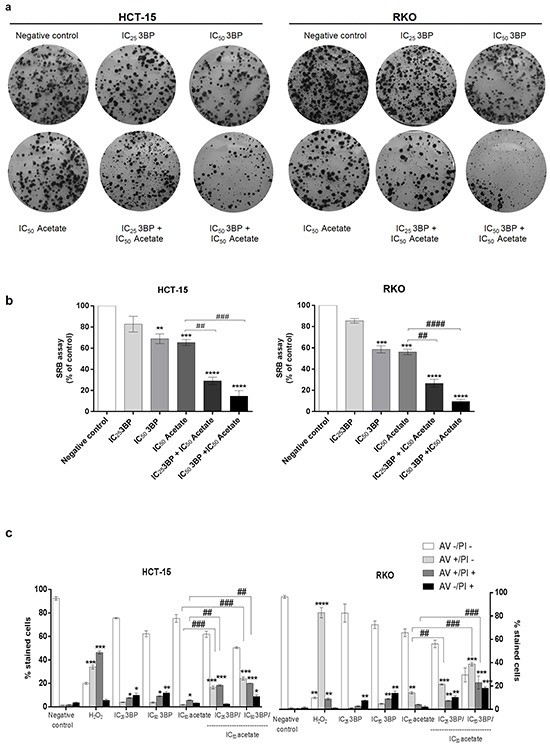
Effect of acetate with 3-bromopyruvate (3BP; a glycolysis inhibitor) in CRC cells **a, b and c.** CRC cells were treated with IC_25_ and IC_50_ values for the 3BP (17.5 uM, 35 uM and 75 uM, 150 uM, respectively for HCT-15 and RKO cells). The treatments were performed with 3BP alone or with acetate treatment combination (only the IC_50_ value for the acetate: 70 mM for HCT-15 and 110 mM for RKO cell line) after 48 h of incubation. 3BP was added 16 hours before to complete 48 hours of the treatment. As negative control, cells were treated with fresh medium. H_2_O_2_ (500 uM or 1 mM for HCT-15 or RKO cell lines, respectively) was used as positive control. **a.** Colony formation assay during 14 days shows that the combined treatment (IC_25_ of 3BP/IC_50_ of acetate and IC_50_ of3BP/IC_50_ of acetate) decreases cell proliferation (number of colony formed at the end of the assay) in both CRC cell lines in a dose-dependent manner compared to the negative control and with the same dose of the 3BP or acetate alone. **b.** Sulforhodamine B (SRB) assay analyzes the cell proliferation of the same conditions. Values represent mean ± SD of at least three independent experiments. **P ≤ 0.01, ***P ≤ 0.001 and ***P ≤ 0.0001 compared with negative control cells and ## P ≤ 0.01 and #### P ≤ 0.0001 comparing acetate alone with combined treatment (IC_25_ of 3BP/IC_50_ of acetate and IC_50_ of3BP/IC_50_ of acetate). **c.** Quantitative analysis of AV/PI staining in HCT-15 and RKO cells. Values represent mean ± SD (AV – PI -, AV + PI -, AV + PI + and AV – PI +) of each condition (n=3). *P ≤ 0.05, **P ≤ 0.01, ***P ≤ 0.001 and ***P ≤ 0.0001 compared with negative control cells and ## P ≤ 0.01 and #### P ≤ 0.0001 comparing acetate alone with combined treatment (IC_25_ of 3BP/IC_50_ of acetate and IC_50_ of3BP/IC_50_ of acetate).

We have previously showed that butyrate at concentration ranging from at 500 – 10.000 μM increased MCTs expression and localization to the plasma membrane sensitizing breast cancer cells to the glycolytic inhibitor 3-bromopyruvate (3BP) [17, 27]. Since acetate, like butyrate exhibited a similar effect in CRC cells regarding the expression of MCT1 and MCT4 and the increased CRC cell glycolytic phenotype, we hypothesized that acetate could also sensitize CRC cells to 3BP. To explore this hypothesis, we studied the combined use of acetate with 3BP. We performed colony formation assays (CFA) in CRC cells treated with 3BP IC_25_ and IC_50_ alone or in combination with acetate IC_50_. We found that the combined treatment (IC_25_ of 3BP/IC_50_ of acetate and IC_50_ of 3BP/IC_50_ of acetate) decreased cell proliferation (number of colonies formed) in both CRC cell lines (Figure 7a). Analysis of cell proliferation by SRB assay (Figure 7b) showed that for all conditions tested, except for the IC_25_ of 3BP, there was a significant reduction in the proliferation of HCT-15 and RKO. The combination of IC_25/_IC_50_ 3BP with the IC_50_ acetate significantly potentiates the effect of acetate *per se* in the inhibition of cell proliferation (p > 0.01 and p > 0.0001, respectively) in both CRC cell lines.

We show that treatment with 3BP alone induced a significant increase (p > 0.05 and p > 0.01) in cells stained with AV/PI in a dose-dependent manner in both CRC cells. In addition, we found that the combined treatment of 3BP and acetate (IC_25_ of 3BP/IC_50_of acetate and IC_50_ of 3BP/IC_50_ of acetate) potentiates apoptosis, as the number of early and late apoptotic cells showed a significant increase in comparison with acetate alone (p > 0.01 and p > 0.001) in both CRC cells. In summary, our results show that 3BP in combination with acetate increased the anti-proliferative effect of acetate and potentiates acetate-induced apoptosis in CRC cells.

## DISCUSSION

Acetate is the main SCFA produced by *Propionibacterium* which normally reside in the human colon. It has been shown that the concentration of SCFA, including acetate, is modulated by numerous factors such as intestinal microbial community, diet, age, medication and intestinal diseases [28, 29]. The colonic SCFA acetate can be found in the gut at considerably high concentrations, which physiological levels range from 40–80 mM, although there is a variation along the human colon and specially after a dietary fiber-containing meal [8, 30].

SCFAs, including acetate, show anti-tumorigenic properties in CRC cells and transformed colon cells, while exerting a protective role in normal colonic crypts [3, 4]. The cellular and molecular mechanisms underlying acetate-induced apoptosis in CRC cells have been studied [5, 9, 31], however the reason for acetate selectivity towards transformed colon and CRC cells is still elusive. To exert its effects and access the cellular targets, acetate has to be transported across the plasma membrane of colon cells. Studies on SCFA in normal colon and CRC cells have been focused mainly on butyrate transport [1, 12, 13], being the information on acetate transport quite scarce.

In an attempt to characterize the transport of acetate across CRC cells, here we carried out kinetics and energetic studies of acetate uptake. We demonstrated that at pH 6.0 (similar to the gut environment), acetate transport across CRC cell membrane is strongly inhibited by monensin and CHC but not by DIDS and AR-C155858, supporting the contribution of SMCT1 in acetate transport in CRC cells. SMCT1 is an active unidirectional transporter that mediates lactate uptake and is expressed abundantly in the apical membrane of the colon [12, 32, 33]. Our results on acetate transport are in accordance with several reports showing that SMCT1 transports monocarboxylic acids such as butyrate, 3BP and dichloroacetate in cancer cells [17, 34–36]. On the other hand, some reports have demonstrated that SMCT1 is silenced in some CRC by DNA methylation [1, 12, 34, 36], conferring a selective advantage to escape butyrate-induced cell death [12, 37]. This could also be true for acetate as here we showed that in CRC cells expressing SMCT1, this transporter is relevant for acetate uptake and consequently for the acetate-induced apoptosis effect in CRC cells.

Acetate was also demonstrated to enter normal colon cells by passive transport [11]. Our experimental data suggest that diffusion through aquaporins, small transmembrane channel proteins [24, 38, 39], also contributes to acetate uptake, since acetate transport was inhibited by HgCl_2_. The contribution of aquaporins to acetate uptake could explain the different kinetics of acetate transport in the CRC cells studied. Indeed, acetate transport follows a first order kinetics in RKO cells, being less affected by CCCP or monensin than HCT-15 cells, likely due to a higher contribution of aquaporins for acetate transport in RKO cells. To the best of our knowledge, this is the first report suggesting that aquaporins, found upregulated in different types of cancer including CRC [22, 24, 40], might play a role in acetate transport in CRC cells.

Monocarboxylate transporters 1 to 4 (MCT 1-4) are proton symporters, involved in the uptake and/or efflux of pyruvate, lactate, ketone bodies and SCFA through the plasma membrane [11, 15]. We investigated if exposure to acetate could regulate its transport through MCTs. Using the inhibitor AR-C155858, we showed, that MCT1 and/or MCT2 participate actively in the transport of acetate across the membrane in CRC cells exposed to physiological doses of acetate. These observations are in accordance with the transport of butyrate into colon cancer cells mainly by MCT1 [1, 13, 41]. Moreover, MCT1 was also reported to be responsible for acetate transport in mouse cancer cells (Ehrlich-Lettre ascites cells) [42].

Despite some controversies in the literature, MCT1, MCT2 and MCT4 are found upregulated in CRC compared to normal epithelium [15, 43, 44]. There are different mechanisms involved in the regulation of MCTs, however their regulation by SCFA (especially acetate) needed further clarification [1, 12, 13, 41]. It has been shown that upon butyrate treatment, MCT1 is the most abundant MCT isoform expressed in the CRC Caco-2 cells [13]. Our data show that exposure to acetate increased the expression of MCT1, MCT4 and the glycosylated CD147, associated with MCT1 re-localization to the plasma membrane of CRC cells. Indeed, MCT1 and MCT4 require association with CD147 for proper plasma membrane localization and function [18]. Our results put forward a possible role of acetate in the regulation of its uptake/transport, both by controlling the expression of MCTs and CD147, with consequent MCT1 functionalization at the plasma membrane in CRC cells.

Most cancer cells, including CRC, exhibit a hyper-glycolytic phenotype, which is characterized by production of high amounts of lactate, contributing to acidification of the tumor microenvironment [15, 45]. As glucose becomes the primary source in CRC cells, glucose transporters (GLUTs) and MCTs have a central role in the maintenance of the cancer cell glycolytic metabolism [14, 46]. Here, we observed that acetate induces expression of both MCT1 and MCT4 over time, without changes in the levels of GLUT1, the main glucose transporter expressed in CRC [16, 41, 47]. Furthermore, upon acetate treatment, we observed an increase in glucose consumption and lactate production up to 24h in CRC cells, which goes in line with the increased expression of MCTs and the need to export the produced lactate [48–51]. Our findings are in agreement with Matthews *et al*, describing that butyrate and propionate, alone or in combination, significantly increased glucose consumption. The authors explained the elevated glucose consumption rates, mediated by an increase in oxidative pentose pathway activity (important pathway of glucose metabolism), as a way to produce energy efficiently [6]. In addition, the increase on glucose consumption and lactate production after acetate treatment may be correlated with our previous reports showing that acetate treatment is associated with mitochondria dysfunctions, such as an increase in mitochondrial mass, an accumulation of superoxide anion and a decrease in mitochondrial membrane potential, even after short incubation times (12 and 24 hours after acetate treatment) [9]. The increase in glucose uptake/consumption by CRC cells after 24 hours of acetate exposure may therefore reflect a response that cells convey to cope with acetate-induced mitochondrial dysfunction and consequently oxidative phosphorylation impairment. Indeed, mitochondria dysfunction induced by acetate might contribute to the observed switch to the glycolytic metabolism up to 24 hours in CRC cells.

Due to MCTs overexpression in cancer and important role in the maintenance of glycolytic metabolism [14, 43], MCTs became attractive targets in cancer therapy, especially in cancers with a hyper-glycolytic phenotype like CRC [14, 46]. However, they can also be used to transport of drugs into cancer cells, behaving as “Trojan horses” [17, 52]. In fact, MCTs are described to mediate the entry of the chemotherapeutic agent 3BP used to selectively kill cancer cells [14, 17]. In this context, it was demonstrated that butyrate mediated-increase in MCTs expression and plasma membrane localization sensitizes breast cancer cells to 3BP [17, 27]. Since acetate exhibited a similar effect on MCT expression in CRC cells, we hypothesized that acetate could also sensitize CRC cells to 3BP. Our results showed that, in comparison to the effect of acetate or 3BP alone, the combination of both compounds is more effective in the inhibition of cell proliferation and induction of apoptosis in CRC cells, which might have important implications in CRC therapy.

Summing up, we show that acetate uptake involves at least two distinct mechanisms of transport in CRC cells namely mediated by SMCT1, MCT1 and/or MCT2 and passive transport by aquaporins. In addition, we found that acetate upregulates MCT1, MCT4 and CD147, with re-localization of MCT1 at the plasma membrane, associated with an increase in the glycolytic phenotype.

Our data showing an important role of MCTs and aquaporins in acetate uptake, consistently with reported data on MCTs and aquaporins overexpression in CRC clinical cases, may underlie acetate selectivity towards CRC cells.

Finally, we identified a novel approach for CRC therapy, based on the elimination of CRC cells exposed to acetate, through their sensitization by 3BP or another glycolytic inhibitor whose transport is mediated by MCTs.

## MATERIALS AND METHODS

### Material

#### Reagents

Sodium acetate and HgCl_2_ were purchased from Merck. Radiolabelled [^14^C] acetate (specific activity of 55.2 mCi/mmol) from PerkinElmer. Carbonyl cyanide m-chlorophenyl hydrazone (CCCP), monensin, valinomycin and α-cyano-4-hydroxycinnamic acid (CHC) from Sigma. 4,4'-Di-isothiocyano-2,2'-stilbenedisulfonic acid (DIDS) was obtained from Santa Cruz Biotechnology and AR-C155858 was a gift from AstraZeneca.

#### Cell lines

Human colorectal cancer (CRC) cell lines, HCT-15 and RKO were obtained from the American Type Culture Collection (ATCC). Cells were cultured at 37 °C under a humidified atmosphere containing 5% CO_2._ HCT-15 cells were grown in Roswell Park Memorial Institute (RPMI) medium and RKO cells in Dulbecco's Modified Eagle Medium (DMEM).

All culture media were supplemented with 10% fetal bovine serum and 100 U/ml penicillin/streptomycin.

### Methods

#### Acetate uptake assay

The protocol for [^14^C] acetate uptake used was described previously [17]. For normalization, protein was quantified using a BCA Protein Assay Kit (Pierce). As [^14^C] acetate uptake was linear up to 5 min, 3 min of incubation was used. The effect of inhibitors: AR-C155858, CHC, DIDS, CCCP, monensin, valinomycin and HgCl_2_ was evaluated in cells incubated for 3 min with each compound in MES buffer, pH 6.0, prior to incubation with 1.0 mM [^14^C] acetate for 3 min.

#### Western blotting assay

Cells were seeded in 6-well plates and exposed to acetate for 48 hours: 45 mM, 70 mM for HCT-15 and 75 mM, 110 mM for RKO cells (IC_30_ and IC_50_ acetate doses for both cell lines) previously determined by us [5]. As negative control, cells were incubated with fresh medium. Cell lysis, total protein and Western blotting were carried out as previously described [5].

The primary antibodies used were: anti-MCT1 (1:500), anti-MCT2 (1:200), anti-MCT4 (1:500), anti-SMCT1 (1:250), anti-CD147 (1:500), and anti-actin (1:5000), from Santa Cruz Biotechnology; anti-GLUT 1 (1:200) (Abcam). Chemiluminescence was detected using the ECL detection system (Amersham) and the imager Chemi-Doc XRS system (Bio-Rad).

#### Immunofluorescence assay

CRC cells were seeded in 12-well plates containing glass coverslips and exposed to acetate during 48 hours (IC_30_ and IC_50_ acetate doses) for the cellular localization of MCT1, MCT4 and CD147. The immunofluorescence protocol used was described previously [53]. At the end, coverslips were mounted on Vectashield mounting medium with DAPI and observed under a fluorescence microscope (Olympus BX61 fluorescence microscope). Three coverslips were prepared for each experimental condition.

#### Determination of glucose consumption and lactate production

Cells cultured in 48-well plates were pre-incubated in glucose-free media for 2 h, cells were washed with PBS and incubated with acetate (IC_30_ and IC_50_, as mentioned before). Conditioned medium of non-treated cells (negative control) or exposed to acetate was collected at 3, 6, 12 and 24 hours. Glucose consumption and extracellular lactate were measured by the enzymatic colorimetric kits: Glucose Assay Kit (Roche, Mannheim, Germany) and Lactate Assay kit (SpinReact) Fluorescence intensity was detected with absorbance emission at 490 nm. Values are expressed as the mean of fluorescence intensity normalized to T0 (control for glucose and lactate levels before the treatment with acetate) and the cell biomass was analyzed by Sulforhodamine (SRB) assay as previously described [5].

#### Sulforhodamine (SRB) assay

The IC_50_ concentrations of 3-bromopyruvate (3BP) were calculated by the SRB assay as previously described [5]. The following 3BP doses were used: 17.5 μM, 35 μM and 75 μM, 150 μM; IC_25_ and IC_50_, respectively for HCT-15 and RKO cells). For the SRB assay with acetate and 3BP, 3BP was co-incubated 16 hours before completing 48 hours of acetate treatment. Briefly, HCT-15 and RKO were seeded in 24-well and incubated 48 hours with 70 mM and 110 mM of acetate (IC_50_ for HCT-15 and RKO cells, respectively) previously determined [5] with and without previous co-incubation with the IC_25_ and IC_50_ doses of 3BP for 16 h. As negative control cells were incubated with fresh medium and as positive control we used H_2_O_2_ (500 mM or 1 mM for HCT-15 or RKO cell lines, respectively). All samples were measured in triplicates and the values were expressed relative to the negative control.

#### Colony formation assay (CFA)

Cells were seeded in 6-well plates at 600 cells/mL, 300 cells/mL (respectively for HCT-15 and RKO cell lines) and were treated as described for the SRB assay (same conditions). After 48 hours of treatment, the medium was replaced by fresh medium twice *per* week during 14 days. To evaluate the colony numbers formed for each condition, cells were washed with PBS and fixed with 6% glutaraldehyde/0.5% crystal violet solution for 30 min, at RT. The cells were then washed with water and air-dried.

#### Apoptosis assay

Cells were seeded in 6-well plates and were treated as described above for the SRB assay (same conditions). The percentage of cells undergoing apoptosis after 48 h of acetate treatment was determined using Annexin-V FITC (AV) (BD Biosciences) and Propidium iodide (PI) (Sigma). Both floating and attached cells were collected and prepared as described previously [5]. Cell viability and cell death was assessed by double staining with Annexin-V FITC/ PI. Unstained and stained cells were classified as follows: viable cells (AV-/PI-), early apoptotic cells (AV+/PI-), late apoptotic cells (AV+/PI+) or necrotic cells (AV-/PI+).

#### Statistical analysis

Kinetic parameters were determined using Prism software version 6 (GraphPad) for the non-linear regression of the values of the initial uptake rates of acetate as a function of the acid concentration. Other statistical significance analysis were determined by two-way ANOVA or one-way ANOVA followed by Dunnett or Bonferroni's test for multiple comparisons. All results are presented as mean ± standard deviation (SD) of three independent experiments. Differences were considered significant for *P* values lower than 0.05.

## Abbreviations

AV: Annexin V
CRC: colorectal cancer
CCCP: carbonyl cyanide m-chlorophenyl hydrazone
CFA: colony formation assay
CHC: a-cyano-hydroxycinnamic acid
DCA: dichloro acetate
DIDS: 4,4'-Di-isothiocyano-2,2'-stilbenedisulfonic acid
IC: inhibitory concentration
MCT: monocarboxylate transporter
PI: Propidium iodide
RT: room temperature
SCFA: short-chain fatty acids
SRB: sulforhodamine B
3BP: 3-bromopyruvate
